# Progress of single-cell RNA sequencing combined with spatial transcriptomics in tumour microenvironment and treatment of pancreatic cancer

**DOI:** 10.1186/s12967-024-05307-3

**Published:** 2024-06-12

**Authors:** Jie Zhu, Ke Zhang, Yuan Chen, Xinyu Ge, Junqing Wu, Peng Xu, Jie Yao

**Affiliations:** 1 Department of Hepatobiliary and Pancreatic Surgery, Northern Jiangsu People’s Hospital Affiliated Yangzhou University, Jiangsu Province, China; 2 Dalian Medical University, Dalian, China; 3 Department of Hepatobiliary and Pancreatic Surgery, Northern Jiangsu People’s Hospital Affiliated Yangzhou University, Jiangsu Province, China

**Keywords:** Pancreatic cancer, Treatment, Single-cell RNA sequencing, Spatial transcriptomics, Tumour microenvironment

## Abstract

In recent years, single-cell analyses have revealed the heterogeneity of the tumour microenvironment (TME) at the genomic, transcriptomic, and proteomic levels, further improving our understanding of the mechanisms of tumour development. Single-cell RNA sequencing (scRNA-seq) technology allow analysis of the transcriptome at the single-cell level and have unprecedented potential for exploration of the characteristics involved in tumour development and progression. These techniques allow analysis of transcript sequences at higher resolution, thereby increasing our understanding of the diversity of cells found in the tumour microenvironment and how these cells interact in complex tumour tissue. Although scRNA-seq has emerged as an important tool for studying the tumour microenvironment in recent years, it cannot be used to analyse spatial information for cells. In this regard, spatial transcriptomics (ST) approaches allow researchers to understand the functions of individual cells in complex multicellular organisms by understanding their physical location in tissue sections. In particular, in related research on tumour heterogeneity, ST is an excellent complementary approach to scRNA-seq, constituting a new method for further exploration of tumour heterogeneity, and this approach can also provide unprecedented insight into the development of treatments for pancreatic cancer (PC). In this review, based on the methods of scRNA-seq and ST analyses, research progress on the tumour microenvironment and treatment of pancreatic cancer is further explained.

## Introduction

Pancreatic ductal adenocarcinoma (PDAC) is a highly malignant neoplasm with a poor 5-year survival rate of less than 5% [1]. To improve the outcomes of treatment strategies for PDAC, it is first necessary to understand how cancer cells develop and spread to neighbouring tissues. The TME plays a crucial role in tumour progression, as cells respond sensitively to changes in local cues [2]. The TME encompasses all noncancerous host cells in tumours, including fibroblasts, immune cells, and endothelial cells. In addition, the TME contains some noncellular components, including extracellular matrix (ECM) and soluble products, such as chemokines, cytokines, and growth factors [3]. All of these cells and their associated secreted factors and molecules have a significant impact on tumour resistance, immune escape, and metastasis [4].

ScRNA-seq plays an important role in furthering our understanding of the TME but the process of sequence analysis of tissue samples eliminates spatial information about cells, which hinders our ability to explore interactions among cells in the tumour microenvironment. In recent years, the problem of loss of spatial information for RNA analytes has been addressed by combining scRNA-seq with ST [5]. With this combination of approaches, we can analyse RNA expression at its native location to enhance our understanding of factors that determine cell morphology, genotype, and the microenvironment, which in turn allows us to further deepen our understanding of the mechanisms of pancreatic cancer development and develop more effective treatments for pancreatic cancer [6].

## Single-cell RNA sequencing and spatial transcriptomics

### Single-cell RNA sequencing (scRNA-seq)

**Fig. 1:**
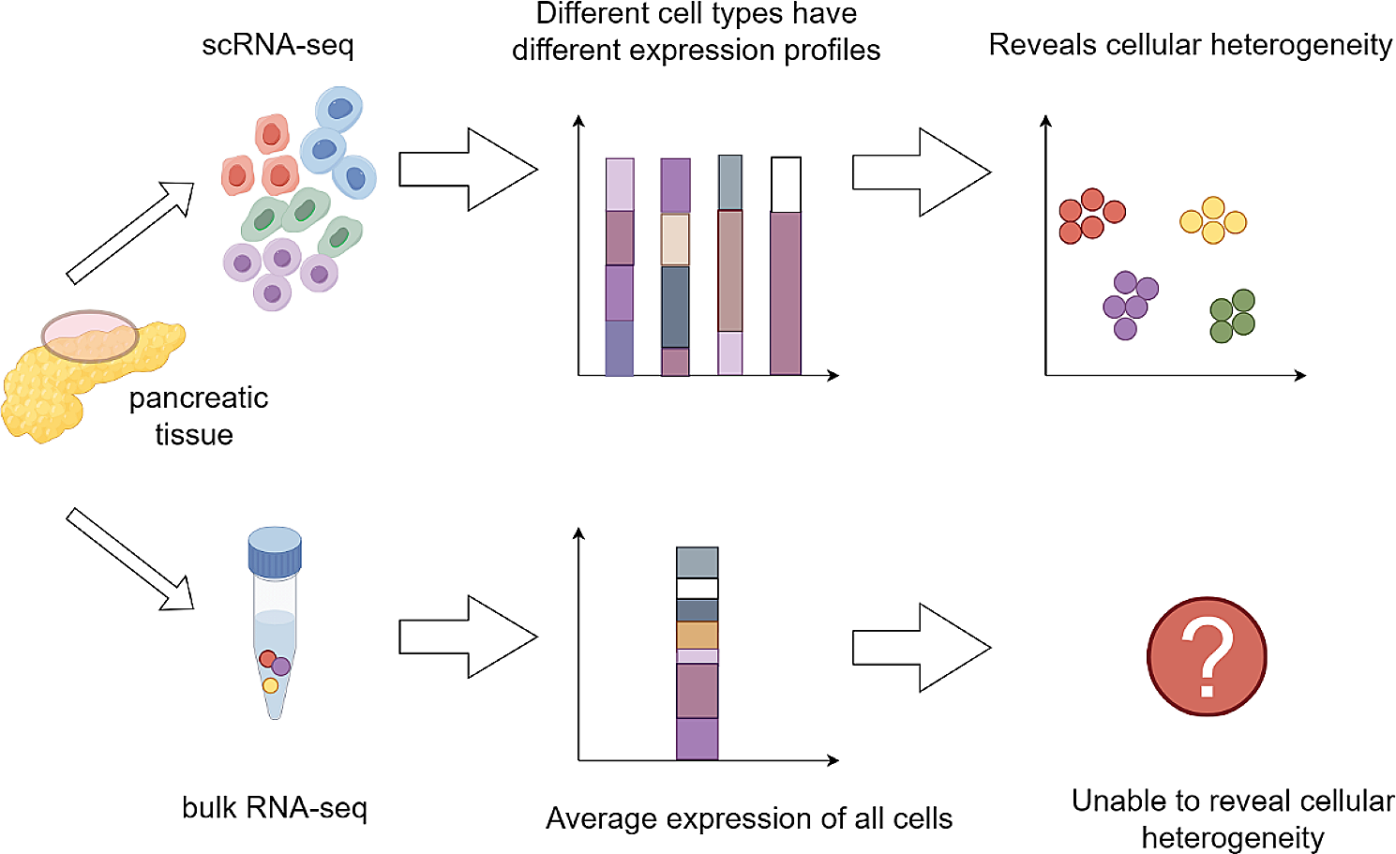
Comparison plot of scRNA-seq and bulk RNA-seq approaches. ScRNA-seq can reveal cellular heterogeneity, while bulk RNA-seq cannot. ScRNA-seq, single-cell RNA sequencing. *Figure 1 was generated with Figdraw

Bulk RNA sequencing is a traditional sequencing method, which mixes RNA together for sequencing by extracting RNA from cells or tissues. The key distinction between Bulk RNA-seq and scRNA-seq lies in the scale and sensitivity of the analysis [7]. Unlike traditional bulk RNA sequencing, scRNA-seq can be used to detect rare or heterogeneous cell populations by analysing individual cells that would otherwise be masked by bulk sequencing approaches (Fig. 1) [8]. Common procedures in scRNA-seq include the isolation of single cells, RNA extraction, reverse transcription, preamplification and detection [9]. In recent years, great progress and breakthroughs have been made in the use of scRNA-seq in cancer research, and its high resolution at the single-cell level allows it to be used to explore rare cell subsets that we previously knew little about as well as the heterogeneity and molecular subtypes of tumour cells, and it is also of great help in the identification of circulating tumour cells (CTCs) and cancer stem cells (CSCs) [10, 11, 12]. In addition, the advent of scRNA-seq has led to a deeper understanding of the TME and the tumour immune microenvironment (TIME), which has reciprocally led to the wider use of scRNA-seq for identifying mechanisms associated with tumour development, progression, metastasis, evolution, recurrence, and treatment resistance [13–16]. At present, several cutting-edge sequencing platforms, including 10× Genomics, have been widely used by researchers. Via scRNA-seq, researchers can now perform high-throughput analysis of thousands of single cells simultaneously and high-resolution analysis of the transcriptome and genome of individual cells [17]. Single-cell sequencing offers numerous benefits compared to conventional bulk sequencing techniques. These advantages include the ability to specifically identify uncommon cell types or subgroups and the ability to uncover genetic and epigenetic changes at the individual cell level. Furthermore, it can provide valuable perspectives on cellular diversity and clonal development within complex tissues and has the ability to identify innovative biomarkers and define therapeutic objectives. Currently, the use of single-cell sequencing has become prevalent in diverse areas of medical research [18]. ScRNA-seq is particularly crucial in the field of cancer research because it can be used to reveal the diversity of cancer cells and track the progression of tumour growth. Furthermore, examining the transcriptomes of immune cells within tumour tissues enables the investigation of immune cell behaviour, including evasion strategies and resistance to drugs. This research is beneficial for advancing the development of improved clinical targeted therapy and immunotherapy approaches. Via analysis at the single-cell level, scRNA-seq enables the exploration of cell‒cell communication and interactions among malignant and nonmalignant cells in the TME, allowing us to gain a comprehensive view of diverse tumour microenvironments [19]. 

However, the high cost of scRNA-seq discourages some researchers when large-scale samples need to be sequenced. To address this issue, the researchers constructed single-cell gene expression profiles by integrating scRNA-seq data and then deconvoluted bulk RNA-seq based on reference profiles. Cell type deconvolution is a computational method to determine the proportion of various cell types from bulk RNA-seq data, and it has now been increasingly used to analyze different cell types in tumor tissues [20, 21]. In summary, researchers can deconvolute RNA-seq bulk samples by referring to smaller scRNA-seq datasets, so as to obtain specificity information for various cells while obtaining transcriptional profiles of a large number of samples [22]. However, deconvolution also has its limitations, mismatched scRNA-seq references or references with inaccurately annotated cells can severely deconvolution performance [23]. Some of the latest online tools developed based on deconvolution algorithms are BayesPrism [24], MuSiC2 [25], TIMER2.0 [26], Bisque [27], Coex [28].

### Spatial transcriptomics (ST)

The development of ST constitutes a breakthrough in the field of medical biotechnology, and it can be used for high-throughput analysis of spatial localization and related analysis of transcripts in biological systems for various applications [29]. Compared with traditional biological studies, ST can provide spatial information at the transcriptome level. This method was first applied to mRNA analytes in 2016, and its increased resolution and increased sensitivity compared to the previously existing approaches rapidly attracted interest [30]. The study of RNA analytes using spatial transcriptomics can be performed with different methods [31]. Overall, spatial transcriptomics methods can be divided into four broad categories [32, 33, 34]: (1) next-generation sequencing (NGS), in which the advances made in sequencing by NGS platforms are obvious, and we can use NGS platforms to restore spatial location data for transcripts by constructing RNA sequencing libraries; (2) imaging-based techniques, including fluorescence in situ hybridization and in situ sequencing-based methods, in which transcripts are analysed using imaging probes sequentially hybridized in tissue [35–37]; (3) a probe-based method that counts barcode probes of known targets to obtain spatial information; and (4) image-guided spatially resolved single-cell RNA sequencing; in which individual cells in a region of interest (ROI) based on microscopy images are selected and isolated prior to single-cell sorting and state-of-the-art single-cell omics sequencing [34–39]. Many spatial transcriptomics methods have been commercially developed, and many platforms have been developed to perform spatial transcriptomics approaches with unique characteristics, each of which has its own advantages in spatial resolution and scope of application [40]. To date, with the in-depth study of spatial transcriptomics, additional commonly used spatial transcriptomics research methods have been developed. Among the related platforms is the 10 × Genomics Visium platform; in 2019, 10 × Genomics launched the Visium spatial transcriptomics platform, which uses microarrays containing spatial barcode oligonucleotides (dT) to capture mRNA from tissues covered on chips prior to processing for sequencing to generated unbiased spatial transcriptomics data [30]. Another mainstream platform for spatial transcriptomics analysis is GeoMx DSP, which was introduced by NanoString. Because of the unprecedented high-throughput and single-cell resolution of these platforms, researchers are better able to explore spatial information at the transcriptome level. In recent years, the development of sequencing technology has been rapid, and sequencing technology itself is also continuously improving [41]. The wide application of single-cell transcriptome analysis is obvious, but it also has an intrinsic disadvantage; that is, all information related to the spatial organization of cells in tissues is permanently lost due to the need for tissue dissociation [42]. In response to the shortcomings of single-cell transcriptomics approaches, spatial transcriptomics reveals information about the spatial distribution of gene expression profiles, which provides researchers with information about tissue characteristics that cannot be obtained by scRNA-seq [43, 44, 45, 46].

## Application of scRNA-seq and ST in pancreatic cancer

### Combined scRNA-seq and ST analysis of cancer-associated fibroblasts (CAFs) in PDAC

In seeking to understand the tumour microenvironment, researchers have found that cell‒cell interactions are an important part of the tumour ecosystem; that is, there are cellular interactions between groups of cells in the same region and that these interactions are involved in certain responses. It is generally accepted that CAFs play an important role in the tumour microenvironment and that they promote PDAC progression [47, 48, 49].

**Fig. 2:**
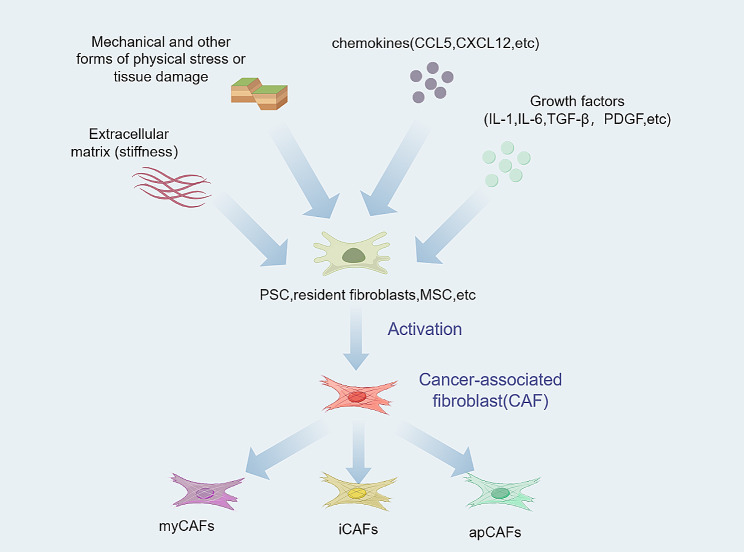
Several factors contribute to the activation of CAFs. Activated CAFs are further divided into different subtypes such as myCAFs, iCAFs, and apCAFs

CAFs can be transformed from a variety of cells, such as pancreatic stellate cells (PSCs), resident fibroblasts, mesenchymal stem cells (MSCs), etc [50–54]. These cells are transformed into CAFs upon stimulation with modulators including cytokines such as IL-6, TGF-β, IGF-1, PDGF, FGF, etc. In addition, some chemokines, such as CCL5 and CXCL12, have also been demonstrated to be involved in the process of CAFs activation. There are other factors, such as the modifications of mechanical properties of the ECM (e.g., stiffness), mechanical and other forms of physical stress or tissue damage [55–57, 58–62] (Fig. 2). Although the role of CAFs in PDAC remains to be further explored, it has already been shown that therapeutic strategies targeting CAFs are feasible [47, 63, 64].

To date, many groups have focused their attention on the interactions among cancer-associated fibroblasts (CAFs) and other types of cells. In the analysis of these interactions, single-cell RNA sequencing has unique advantages and can help us gain a better understanding of different cell subsets. In a recent cross-species single-cell analysis of PDAC, Elyada et al. confirmed the presence of myofibroblastic CAFs (myCAFs) and inflammatory CAFs (iCAFs) using single-cell RNA sequencing and discovered a new CAF population, i.e., “antigen-presenting CAFs” (apCAFs) (Fig. 2). By analysing the genetic profiles of these subsets, they found that the iCAF subcluster expressed high levels of the lectin Clec3b as well as chemokines and inflammatory mediators such as IL-6, Cxcl1, and Ly6c1. The myCAF subcluster exhibited robust expression of the smooth muscle-specific genes Acta2 and Tagln, along with Igfbp3, Thy1, Col12a1, and Thbs2. The newly defined apCAF subcluster showed unique upregulation of pathways related to antigen presentation and processing, fatty acid metabolism, MYC targets, and MTORC1 signalling [65, 66]. The discovery of the different subpopulations of CAFs and differences in the function of each subpopulation constitutes an immense breakthrough in the study of the PDAC tumour microenvironment, indicating the possibility of a precision PDAC therapy targeting CAFs [67]. 

However, the loss of spatial information remains an inevitable limitation of scRNA-seq. By combining scRNA-seq with ST, we can gain an understanding of the functional interactions of cells and how they differ among tissue regions. Some groups have defined the transcriptomes of cancer-associated fibroblasts (CAFs) proximal and distal to tumours by combining spatial transcriptomics and single-cell RNA sequencing (scRNA-seq) datasets and linking the transcriptome data to clinical outcomes. These studies showed that the tumour-proximal CAF population contained numerous myofibroblastic CAFs (myCAFs) with robust expression of podoplanin and Wnt ligand signalling. In contrast, inflammatory CAFs (iCAFs) dominated the tumour-distal subset of CAFs and expressed complement components and the Wnt inhibitor SFRP2. Although poor clinical outcomes are associated with elevated HIF-1α and podoplanin expression, inflammation and the expression of complement genes are predictors of prolonged survival [68]. This ability to analyse the spatial distribution of different subtypes of CAFs is a unique advantage of ST that has allowed us to determine that the transcriptional activity of different subsets of CAFs is strongly regulated by their relative proximity to tumours.

An ST analysis by Ren et al. revealed that fibroblasts in the stroma of tumour-adjacent tissue exhibit tumour-promoting effects and generate a tumour-limiting state, and FGG + CRP + inflammatory fibroblasts associated with cancer-replaced islets in pancreatic cancer were first discovered in this ST analysis [69]. In addition, Moncada et al. developed a cross-modal analysis method (MIA) for integrated analysis of scRNA-seq and ST data. They defined three gene expression modules in PDAC cancer cells by analysis of scRNA-seq datasets: the hypoxia response, oxidative phosphorylation and stress response modules. Moreover, they used MIA to show that inflammatory fibroblasts and cancer cells expressing stress response gene modules exhibit regional colocalization [70]. This finding provides a new understanding of the role of inflammatory fibroblasts in PDAC tumours in the tumour microenvironment. It is evident that ST is a powerful complement to scRNA-seq. Combining ST and scRNA-seq analyses can reveal a more complete landscape of the TME.

### Application of scRNA-seq and ST in studies of the hypoxic tumour microenvironment of PDAC

Hypoxia is an important feature of solid tumours, including PDAC. It is closely associated with tumour invasion, metastasis, and drug resistance. Many previous studies have shown that various molecules and signalling pathways activated by hypoxia may contribute to the induction of the malignant phenotype of PDAC, which has a great impact on promoting tumour proliferation and invasion [71, 72]. Targeting hypoxia-activated molecules and signalling pathways may constitute a new treatment approach for refractory PDAC. The application of scRNA-seq in the study of tumour heterogeneity and novel cell subsets in PDAC has provided new insights into therapeutic strategies for the development of new targeted therapies [73–75]. However, the use of scRNA-seq for the evaluation of endothelial cell heterogeneity remains limited. Chen et al. performed transcriptomic analysis of endothelial cells in PDAC by scRNA-seq and found that the DEGs in PDAC compared with normal pancreatic tissue were associated with hypoxia and angiogenesis. By further investigating the effects of exosomes on endothelial cells, it was later found that exosomes released from pancreatic cancer cells under hypoxic conditions promote angiogenesis through the miR-30b-5p/GJA1 pathway [76, 77]. Although the advantage of scRNA-seq lies in its intrinsically high single-cell resolution, it is a very important approach for the mechanistic exploration of the hypoxic microenvironment of PDAC.

Hypoxia-inducible factor 1α (HIF-1α) is a potent regulator of the transcriptional response to hypoxia homeostasis and is significantly overexpressed in pancreatic cancer [78]. HIF-1α promotes transcriptional activation in a variety of ways, including activating metabolic switches that promote glycolysis and the expression of various proangiogenic cytokines, including VEGF-A, which acts on surrounding endothelial cells to induce the formation of new blood vessels [79]. Current evidence suggests that HIF-1α can promote tumour progression and distant metastasis and that it is also strongly associated with poor prognosis in pancreatic cancer patients [78]. Maruggi et al. constructed a mouse xenograft model of pancreatic cancer with HIF-1α silencing, and by scRNA-seq, they found that hypoxic cancer cells inhibited glycogenolysis, which promoted glycogen accumulation and promoted the secretion of inflammatory cytokines such as interleukin 1β (IL-1β) and 8 (IL-8). In addition, scRNA-seq analysis revealed enrichment of two bone marrow dendritic cell (cDC) subsets that secrete proangiogenic cytokines, i.e., the cDC1 and cDC2 subsets. This finding suggested that glycogen accumulation associated with the clear cell phenotype in hypoxic cancer cells lacking HIF-1α could activate alternative pathways through which cytokines and DCs could drive angiogenesis [80]. This was an entirely novel finding that revealed that even pancreatic cancer cells lacking HIF-1α have proangiogenic effects under specific conditions. Ref-1 is a redox signalling protein that can regulate the transition of HIF-1α from an oxidized to a reduced state, thereby increasing its ability to bind DNA [81]. Gampala et al. investigated the effects of Ref-1 on metabolic pathways under hypoxic conditions by integrated analysis of scRNA-seq data and identified significant transcriptional variations in genes related to central metabolism, apoptosis, and immune responses, as well as in genes downstream of a series of signalling pathways, in Ref-1 knockdown cells compared to scrambled control cells [81].

However, tumour spatial heterogeneity remains very difficult to investigate by scRNA-seq, and the ability of spatial transcriptomics to provide spatial information allows us to obtain a more in-depth understanding of the tumour microenvironment under hypoxic conditions at the spatial regional level. Elyada et al. used ST to analyse the relationships between prognosis and gene transcriptional characteristics at the spatial level in patients with pancreatic cancer. They found that patients with poor prognosis exhibited ubiquitous PDPN expression in the tumour microenvironment, whereas HIF-1α and VEGF expression also extended into the distal stroma and immune regions, possibly indicating more extensive hypoxia in this patient subgroup [82]. Previous studies showed that HIF-1α expression reflects the hypoxic environment of PDAC tumours and that the degree of hypoxia is an independent determinant of clinical outcome [83, 84]. The results of ST analyses suggest that, from a spatial distribution perspective, the spatial extension of HIF-1α expression into the distal stroma and immune microenvironment is an additional risk factor, indicating that the extent of hypoxia is important for the prognosis of PDAC patients. In a study conducted by Sun and his colleagues to analyse the tumour microenvironment of pancreatic cancer under hypoxic conditions using spatial transcriptomics [85], subsets of tumour cells were found to be changed under hypoxic conditions, that these new cell subsets exhibited spatial and gene expression characteristics different from the original characteristics, and that the expression of the hypoxia-related genes LDHA, TPI1 and ENO1 induced changes. The authors concluded that the hypoxic microenvironment induced changes in pancreatic cancer heterogeneity and the generation of new functional subsets of tumour cells [86]. The new functional subsets may be responsible for PDAC cell survival, proliferation and invasion under hypoxic stress. The Sun group was the first to characterize the changes in PDAC spatial heterogeneity induced by the hypoxic microenvironment and to highlight the latent intercellular communication network under different hypoxic conditions [85]. In addition, the expression and spatial distribution of the hypoxia marker genes HIF-1α and MIF in PDAC xenografts were investigated by ST, and the spatially resolved expression profiles and locations of the HIF-1α and MIF genes showed approximately the same expression levels and overlapping locations, verifying that hypoxia is an effective inducer of MIF expression in PDAC [87]. ScRNA-seq and ST facilitate the exploration of the pancreatic cancer tumour microenvironment under hypoxic conditions at the single-cell and spatial levels, revealing the landscape of the TME in a wider dimension.

### Tumour immunity in PDAC

The tumour immune microenvironment (TIME) is a complex ecosystem composed of various types of cells, such as tumour cells and immune cells, and other cellular components. All the cells have specific connections and interact with each other [88]. Changes in the TIME can induce the progression of cancer and impact the effectiveness of cancer treatment [89]. Relevant studies have shown that changes in the level of T-cell infiltration and the abundance of tumour-associated macrophages (TAMs) can have different effects on the prognosis of patients [90]. In addition, differences in the expression and mutation of PD-1 and PD-L1 in the TIME and differences in the drug response of malignant cells among patients may be related to differences in the efficacy of immune checkpoint blockade (ICB) therapy [91, 92]. PDAC, on the other hand, is highly resistant to immunotherapy, a characteristic thought to be largely related to the impairment of CD8 + T-cell infiltration and activation in tumours [93]. Yousuf et al. demonstrated dysregulation of CD8 + T and natural killer T-cell functions in the TIME of PDAC based on analysis of scRNA-seq datasets. Furthermore, to explore the spatial arrangement of different types of immune cells in PDAC and their correlations with cancerous epithelial cells, this group conducted ST analysis on tissue sections. The findings indicated that there were notable variations in the presence of monocytes, C2Q + macrophages, and CD1 + CD4 + T cells between the basal-like and classical regions. C1Q + macrophages were concentrated in close proximity to basal-like cancerous cells. CD4 + CD52 + T cells were plentiful in basal-like areas but absent from classical tumour sites, consistent with the observed heightened aggressiveness of basal-like tumours [93, 94, 95]. These findings provide additional clarification of the spatial composition of the immune microenvironment in PDAC and its correlation with the local transcriptional state in epithelial cells.

In their study, Yousuf and his colleagues examined cell-to-cell communication using scRNA-seq data. They found that TGFB1-expressing B cells have stronger interactions with CD8 + T cells and macrophages than with other cell types in the TIME. Notably, tumour-infiltrating naive B cells in PDAC showed significantly increased levels of TGFB1 expression, despite the limited presence of naive B cells in PDAC [93, 95]. In a PDAC study by Aziz et al., using GeoMx DSP technology, B cells were found to infiltrate tumours in regions near T cells in long-term survivors, and the abundance of B cells in the tumour region was associated with the infiltration of T cells and APCs, suggesting that CD20 + cells may act as APCs. In addition, relevant T-cell subsets in long-term survivors showed an activated effector phenotype (highly correlated with HLA-DR and CD45RO expression) [96]. GeoMx DSP technology, has unique advantages in the study of differences in immune infiltration at specific locations. The role of B cells in PDAC is not fully understood [97], and in recent years, increasing evidence has suggested that B cells are critical regulators of tumour-induced immune responses [98]. In a study by Meng et al., B cells were shown to be an important component of the tumour-infiltrating lymphocyte (TIL) population in PDAC [99]. Castino et al. reported that both the scattered infiltration of B cells and their organized presence in PDAC tertiary lymphoid structures was associated with improved survival outcomes [100]. Related studies of B cells in PDAC have attracted increasing interest, as scRNA-seq can show the role of B cells in the TIME and their interactions with various other types of cells at the cellular level, while ST can reveal the number and location of B cells in PDAC tissues at the spatial level [95]. 

### Microenvironmental characteristics of precancerous lesions in PDAC

Pancreatic cancer is a highly malignant tumour, and its precursor lesions include pancreatic intraepithelial neoplasia (PanIN), intraductal mucinous neoplasms (IPMNs), intraductal tubular neoplasms (ITPNs), and mucinous cystic neoplasms (MCNs). These precancerous lesions have the potential to contribute to the development of PDAC as cellular and molecular alterations accumulate [101]. 

PanIN is the most common precancerous lesion of PDAC, and it can be found in more than 80% of patients with invasive pancreatic cancer [102, 103]. Given high possibility that PanIN may undergo malignant transformation to PDAC, an increasing number of researchers are investigating PanIN progression. To further understand the composition of PanIN and its microenvironment, scRNA-seq analysis of pancreatic cancer donor organs was performed by Carpenter et al. By comparison of PanIN with tumour samples, they found that both samples exhibited inflammatory gene expression profiles of macrophages. In contrast, the expression profiles of fibroblasts in PanINs were clearly divergent from those in tumours. In addition, they found that T cells were rare or undetectable in normal regions of the pancreas, exhibited a limited presence in PanINs, and were abundant in pancreatic cancer tissues. This analysis revealed similarities and differences in components of the microenvironment among normal pancreatic tissue, pancreatic cancer tissue, and PanIN [104]. When they compared the transcriptional characteristics of PanINs with those of normal acinar and ductal cells, tumour-associated PanINs, and tumour cells in healthy pancreas tissue, they found that accurate identification of PanIN cells in samples was a considerable limitation because of the loss of spatial information during the processing of tissues for scRNA-seq. To address this issue, they leveraged ST, which has the advantage of the ability to identify tissue types at different locations and provide information about the spatial distribution of gene expression profiles [105]. By combining transcriptomic features from spatial data and datasets generated by single-cell RNA sequencing, Carpenter et al. found that the transcriptomes of PanIN cells from normal pancreatic tissue are highly consistent with those of PanIN cells from pancreatic tumour tissue and, most importantly, that PanINs are transcriptionally closely related to pancreatic cancer [104], which may indicate that neoplastic pathways are activated early in tumorigenesis.

Intraductal mucinous neoplasms (IPMNs) are cystic lesions of the pancreatic ductal epithelium that exhibit varying degrees of cellular atypia; they are among the most common precancerous lesions of PDAC and have the potential to progress to pancreatic cancer [106]. Associated data show that progression from IPMN to PDAC accounts for 15–20% of all pancreatic cancer (i.e., PDAC) cases [107]. Therefore, it is important to investigate the mechanism by which IPMN progresses to pancreatic cancer and to explore related interventions. Via ST, Eckhoff et al. found that T cells were the dominant CD45 + cells within the IPMN stroma (56%) and that most of the infiltrating immune cells in IPMNs were T cells and macrophages. Compared to regions of low-grade dysplasia (LGD), regions of high-grade dysplasia (HGD) appear to be rich in macrophages and relatively depleted of T cells. These findings are supported by the transcriptional signatures of proinflammatory macrophages in HGD regions. Although it lacks single-cell resolution, ST is the best approach to analyse the TME in IPMNs [108]. In addition, Sans et al. identified the transcription factor NKX6-2 as a key determinant of gastric cell identity in low-grade IPMNs by ST analysis of the epithelial and microenvironmental characteristics of IPMNs [109]. A previous study showed that the gastric foveal epithelium is one of the most common sites of IPMNs [110]. Thus, Sans et al. identified NKX6-2 as an entirely novel transcription factor that could drive indolent gastric differentiation in IPMN pathogenesis, which in turn led to the development of PDAC [109]. Without single-cell resolution, it is difficult to detect gene expression in low-density cell populations via ST, but this high-resolution spatial analysis method can reveal new ideas for the study of IPMNs. In the future, the combined application of ST and scRNA-seq in the exploration of the mechanisms and treatment of IPMNs is certain.

### PDAC metastasis based on the tumour microenvironment

Pancreatic cancer (PC) is a highly malignant cancer, and approximately 90% of PCs are PDAC [111]. PDAC has a great ability to metastasize to other tissues and organs, which is strongly related to the very poor prognosis of pancreatic cancer patients [112, 113]. Therefore, it is critical to assess the risk of metastasis in patients with pancreatic cancer, explore the mechanism of metastatic invasion, and determine the heterogeneity of pancreatic cancer tumours.

The TME plays a critical role in tumour progression, metastasis and drug resistance, and it contains various cells, such as fibroblasts and immune cells, as well as other noncellular components, including the extracellular matrix. The high resolution of scRNA-seq at the single-cell level is of great help in studying PDAC tumour cells and various cells in the tumour microenvironment that are involved in PDAC metastasis. A recent single-cell-based analysis showed that different cell populations, including cancer cells, fibroblasts, endothelial cells, and immune cells, exhibit distinct phenotypic and transcriptional profiles at the metastatic stage of PDAC compared to their profiles in primary pancreatic tumours [114]. Based on scRNA-seq analysis and functional enrichment analysis of genes, Tang et al. found that HMGB3 is a hub gene associated with EMT in CTCs, the formation of CTC clusters, and infiltration patterns of immune cells promoting tumour progression and metastasis to distant sites [115]. Lin et al. used scRNA-seq-based analysis methods to quantitatively evaluate cell types and statuses within PDAC primary tumours and metastatic lesions to understand their heterogeneity and complexity. It was found that the cellular landscape of PDAC metastases may not be as complex as that of primary tumours, and few cancer-associated fibroblasts were found in metastatic tumour tissues [116]. These results indicate that various types of cells in the PDAC tumour microenvironment are strongly associated with PDAC metastasis.

ScRNA-seq has a major role in the analysis of the tumour microenvironment at the single-cell level, and in a recent study, scRNA-seq was combined with ST to investigate tumour cells with metastatic characteristics in PDAC. Chen et al. investigated the predominant cell types in PDAC by analysing scRNA-seq datasets of PDAC and proposed the scMetR method [7] to assess the risk of metastasis of tumour cells. Some features associated with metastasis were identified by functional enrichment analysis of differentially expressed genes. In addition, to explore the spatial characteristics of metastasis-featuring tumour cells (MFTCs), Chen et al. performed ST analysis and found that metastasis-related genes were highly expressed in cells in the ductal epithelial region and that MFTCs were distributed mainly in the ductal epithelial region [113]. In a previous report, Moncada et al. concluded that inflammation-associated ductal cells were enriched in ductal epithelial areas by the MIA method for integrated ST and scRNA-seq analysis [96, 7]. According to the relevant findings of the analysis, both tumour cells with metastatic features and inflammation-associated ductal cells were enriched in the ductal epithelial region, and PDAC metastasis-associated genes were also highly expressed in this region, possibly indicating that inflammation can promote PDAC metastasis. In a review, Padoan et al. also described the association of inflammatory cells with PDAC progression [117]. Perineural invasion (PNI) is a phenomenon in which cancer cells invade the perineural space, and the presence of PNI often predicts local recurrence and metastasis. Weitz et al. provided novel insights into the aetiology and initiating cues of PNI development in PDAC through DSP [105]. With the further application of ST analysis of the TME, changes in the PDAC microenvironment during metastasis progression can also be identified, which is highly important for studying PDAC metastasis.

## Application of scRNA-seq combined with ST for guiding PDAC therapy

Pancreatic cancer is a malignant tumour of the digestive tract with an insidious onset, rapid progression, very poor therapeutic outcomes and poor prognosis, and its morbidity and mortality are significantly increasing worldwide. When tumours are detected early, surgical resection is still the best approach for curative treatment; however, surgical resection alone is not effective for the vast majority of patients. Surgical resection is often combined with chemotherapy and/or radiation therapy [118]. At present, in addition to surgical treatment, nonsurgical treatments such as chemotherapy, radiotherapy and immunotherapy are available. At present, the commonly used chemotherapeutic drugs include gemcitabine, fluorouracil and albumin-paclitaxel [119]. The tumour microenvironment may weaken immune-related therapeutic effects but enhance the effects of adjuvant therapy, and the current understanding of the spatial structure of the tumour microenvironment and the multicellular interactions occurring therein remains limited. Recent advances in the integration of scRNA-seq with ST will greatly aid in exploring undiscovered biomarkers involved in tumour development, as well as undiscovered antitumor drugs, and will pave the way for better treatment outcomes [120]. 

### Chemoresistance in PDAC

Systemic chemotherapy is mandatory in the treatment of patients with pancreatic cancer, regardless of whether the patient is also treated surgically. It has been demonstrated that the combination of fluorouracil, leucovorin calcium, irinotecan, and oxaliplatin (FOLFIRINOX), as a first-line therapeutic regimen for patients with metastatic pancreatic cancer, results in a longer overall survival time than gemcitabine therapy [82, 45]. Conroy et al. showed that adjuvant therapy with the modified FOLFIRINOX regimen resulted in a significantly longer survival time but a greater incidence of toxic effects than gemcitabine in patients with resected pancreatic cancer [121]. However, pancreatic ductal adenocarcinoma (PDAC) is somewhat resistant to a variety of chemotherapeutic agents (e.g., gemcitabine, 5-fluorouracil, paclitaxel, and oxaliplatin), and this resistance is one of the leading causes of PDAC treatment failure [122]. Therefore, gemcitabine resistance has always been a topic of great concern, and we urgently need to find new treatments to solve the problem of resistance to chemotherapeutic drugs, including gemcitabine.

ScRNA-seq, with high resolution at the single-cell level, can be used to screen pancreatic cancer cell lines and makes it possible to identify gemcitabine-resistant (GR) cells. In a recent study, Principe et al. used scRNA-seq to identify a subset of gemcitabine-resistant tumour cells with robust activation of calcium/calmodulin signalling. In addition, enrichment analysis of differentially expressed genes revealed that calcium signalling-related genes were upregulated in GR cells. Moreover, single-cell RNA sequencing revealed that impaired activation of the RAS/ERK signalling pathway resulted in rapid loss of the resistance phenotype in vitro. In addition, they found that CCBs inhibited pro-survival ERK signalling in vitro and significantly enhanced the response to gemcitabine treatment in orthotopic xenograft and transgenic models of PDAC. These results suggest that CCBs may provide clinical benefit for PDAC patients who develop resistance to gemcitabine [123]. In addition, using scRNA-seq, Cui et al. validated three types of CAFs—iCAFs, myCAFs, and apCAFs—in the CAF population, consistent with the findings of Elyada et al. [65]; however, Cui further classified two iCAF subsets into CD133 + and CXCR4 + subsets. In patients treated with gemcitabine and paclitaxel, they observed upregulation of the metallothionein gene in iCAFs. Metallothionein expression is associated with resistance to multiple chemotherapeutic agents and may indicate the activation of chemoresistance mechanisms [124].

William et al. constructed a high-resolution molecular landscape of the cellular subtypes and spatial communities that compose PDAC using single-nucleus RNA sequencing (snRNA-seq) and whole-transcriptome DSP. This study was performed to reveal possible interactions among malignant cells, CAFs, and immune compartments that promote therapeutic resistance. Spatially defined receptor‒ligand (RL) pairs were coexpressed across ROIs in both CRT-treated and untreated samples. Although some RL pairs were well correlated in both the untreated and CRT specimens, many pairs were differentially correlated by treatment status. Therefore, the RL pairs identified in their study may be important to further investigate in future studies, and a subset of intercellular RL-enriched interactions in treated specimens may facilitate therapeutic resistance and serve as candidates for intervention [125]. 

### Limitations of immunotherapy in PDAC

Immunotherapy, particularly the use of antibody(Ab)-mediated PD-1 and PD-L1 blockers, has led to unprecedented durable clinical responses in various tumour types with positive or negative PD-L1 expression. PD-L1 is expressed in 19–57% of human pancreatic cancers [116]. Little is known about the mechanisms of resistance to immunotherapy in pancreatic cancer, which is a critical knowledge gap in pancreatic cancer research and severely hinders the development of new immunotherapeutic strategies. Despite rapid advances in recent years, the lack of appropriate models for tumours sensitive to αPD-1 immunotherapy remains a major obstacle to progress in immunotherapy for pancreatic cancer [115]. ScRNA-seq revealed two subtypes of TAM (TAM1 and TAM2) [126, 127]. Zhou et al. developed four orthotopic PC mouse models with different PC cell lines. They aimed to characterize PC responses to anti-PD-1 immunotherapy. Eventually, they found that there was a significant increase in the number of TAM2 and a significant increase in the expression of ARG-1, a marker of M2 macrophages, in mouse models that did not respond to αPD-1 therapy. We may conclude that PC tumours resistant to αPD-1 may have promoted macrophage to transform to M2 TAM, which may contribute to tumor resistance to immunotherapy [128–126, 130]. This is likely to be one of the mechanisms mediating resistance to αPD-1 immunotherapy in pancreatic cancer, and therefore, the development of therapeutic approaches targeting TAMs may hold promise for improving the unsatisfactory responses to immune checkpoint inhibitor therapy. In addition, in a study conducted by Pan et al., scRNA-seq revealed that anti-CD47 treatment induced changes in TAMs and upregulated the expression of immune checkpoint receptors such as PD-1 in effector T cells. In animal experiments, they also found that combination therapy targeting CD47 and PD-L1 could promote PDAC growth [131]. These scRNA-seq-based studies revealed changes in TAMs in the tumour immune microenvironment, demonstrated the relationship between alterations in TAMs and immunotherapy resistance in PDAC, and suggested new approaches for TAM-targeting therapies.

In an ST data-based study, Yang et al. first identified bridging genes with high and low expression during malignant transformation initiated by pancreatitis by GeoMx DSP technology, and data from this analysis were combined with RNA-seq datasets to construct the “m7G score” model. They identified FN1 and ITGB1 as core genes in the m7G score model and found that FN1 and ITGB1 can also inhibit T-cell activation by increasing the infiltration of macrophages and neutrophils, which leads to immune escape of pancreatic cancer cells and reduces the response rate to immune checkpoint inhibitor (ICI) therapy [132]. This study suggested that m7G target genes, including FN1 and ITGB1, have the potential to be novel therapeutic targets for PDAC and increase the efficacy of ICIs.

## Conclusion

It is well-known that mutations in PC are driven by genomic mutations. Mutations in PDAC mainly occur in KRAS, TP53, CDKN2A and SAMD4. Of course, there are many genes that are gradually being recognized and studied such as BRCA, APOBEC, KDM6A, etc. [133, 134] Although the links between these genetic mutations and TME have not been clearly elucidated by researchers, related studies have suggested that there is an inextricable link between these two. For example, SAMD4 has an important impact on the development of PDAC by mediating the TGF-β pathway. The TGF-β SMAD4 signalling pathway mediates the tumour-stroma interaction. TGF-β secreting CAFs are involved in inducing epithelial-mesenchymal transformation (EMT) and switching the PDAC proliferative phenotype, which leads to PDAC heterogeneity [135, 136]. This suggests that genetic mutations in tumours not only control PDAC progression, but also have an impact on the metabolic phenotype of cells in the TME.

ScRNA-seq and ST are in a rapidly developing stage, especially ST, which has been increasingly used in tumour studies in recent years, and these novel powerful techniques are of great help in exploring the TME and tumour heterogeneity. To date, scRNA-seq has been widely used to study tumour biology and because of its ability to detect cellular and microenvironmental heterogeneity at single-cell resolution, it has great advantages over traditional sequencing techniques. It can be used to reveal the transcriptome profiles of cancer cells with malignant heterogeneity, characterize gene expression profile dynamics during tumour progression and identify novel subsets, cellular states, and phenotypic transitions [137, 46, 40, 5]. 

CAFs are an abundant component of the tumour stroma and have received considerable attention due to their ability to promote tumour growth and metastasis, interfere with drug delivery, and increase fibroplasia and immunosuppression. However, some studies have suggested that CAFs are associated with improved outcomes in patients with PDAC [138–140]. The differences in these findings may be related to the heterogeneity of CAFs; that is, different CAF subsets may have different functions. Information on the different CAF subsets obtained via scRNA-seq is of great help for further understanding their functions in the tumour microenvironment of pancreatic cancer. Although ST has the advantage of allowing analysis of the spatial distribution of CAFs in these different subsets, relevant ST analyses have revealed that transcriptional activity in the different subsets of CAFs is strongly regulated by their relative proximity to tumours. The study by Ren and Moncada et al. provided a new understanding of the role of inflammatory fibroblasts in PDAC tumours in the tumour microenvironment.

Hypoxia is closely associated with tumour invasion, metastasis, and drug resistance. Various molecules and signalling pathways activated by hypoxia may contribute to the induction of the malignant phenotype of PDAC, which has a great impact on promoting tumour proliferation and invasion. ScRNA-seq and ST help us explore the microenvironment of pancreatic cancer under hypoxic conditions at the single-cell and spatial levels, respectively, providing insight into the landscape of the tumour microenvironment in a wider dimension.

Immunological analysis of pancreatic tumours is key to understanding the progression of pancreatic cancer and further exploring resistance to PDAC immunotherapy. By using scRNA-seq and ST, we can fully understand the heterogeneity of immune cells and explore the distribution and function of T cells, B cells and other immune cells.

In addition, recent studies have shown that the use of scRNA-seq and ST is very important in the study of precancerous lesions and the metastasis of pancreatic cancer. This ability is important for exploring the mechanisms of pancreatic cancer progression, metastasis and invasion.

Pancreatic cancer is a malignant tumour of the digestive tract with an insidious onset, rapid progression, very poor therapeutic outcomes and poor prognosis, and its morbidity and mortality are significantly increasing worldwide. Recent advances in the integration of scRNA-seq with ST will greatly contribute to the exploration of undiscovered biomarkers involved in tumour development, as well as undiscovered antitumor drugs, and will pave the way for better therapeutic outcomes. The use of scRNA-seq and ST has gradually improved our understanding of tumour biological characteristics, and progress in using these techniques in pancreatic cancer research will lead to the identification of more precise potential therapeutic targets for treating PDAC in the future.

In summary, PDAC is an extremely aggressive cancer with a dismal prognosis. By designing rational therapeutic strategies, we can potentially conquer this persistent disease by gaining an understanding of cell-specific characteristics, spatial connections among diverse cells, and time-dependent changes in tumour growth and treatment responses. Although it is in an early stage compared to its progress in the context of other prevalent cancers, precision oncology for pancreatic cancer is anticipated to substantially advance with the combination of scRNA-seq and ST analyses discussed in this review. This will also lead to improved exploration and refinement of adjuvant therapy approaches for PDAC.

## Data Availability

Not applicable.

## Abbreviations

PC: pancreatic cancer
TME: Tumour microenvironment
scRNA-seq: Single-cell RNA sequencing
ST: spatial transcriptomics
PDAC: Pancreatic ductal adenocarcinoma
ECM: Extracellular matrix
CTC: Circulating tumour cells
CSC: Cancer stem cells
TIME: Tumour immune microenvironment
NGS: Next-generation sequencing
ROI: Region of interest
CAF: Cancer-associated fibroblast
iCAF: inflammatory cancer-associated fibroblast
myCAF: myofibroblastic cancer-associated fibroblast
apCAF: antigen-presenting cancer-associated fibroblast
DC: Dendritic cell
VEGF: Vascular endothelial growth factor
PD-1: Programmed cell death protein-1
TAM: Tumour-associated macrophage
ICI: Immune checkpoint inhibitor
snRNA-seq: Single-nucleus RNA sequencing
CCB: Calcium Channel Blocker
MFTC: Metastasis-characteristic tumour cell
PanIN: Pancreatic intraepithelial neoplasia
IPMN: Intraductal mucinous neoplasm
ITPN: Intraductal tubular neoplasm
MCN: Mucinous cystic neoplasm
TIL: Tumour-infiltrating lymphocyte
ICB: Immune checkpoint blockade
EMT: epithelial-mesenchymal transformation
FGF: fibroblast growth factor
PDGF: platelet-derived growth factors
PSC: pancreatic stellate cells
MSC: mesenchymal stem cells
